# Reg IV Is a Direct Target of Intestinal Transcriptional Factor CDX2 in Gastric Cancer

**DOI:** 10.1371/journal.pone.0047545

**Published:** 2012-11-02

**Authors:** Yutaka Naito, Naohide Oue, Takao Hinoi, Naoya Sakamoto, Kazuhiro Sentani, Hideki Ohdan, Kazuyoshi Yanagihara, Hiroki Sasaki, Wataru Yasui

**Affiliations:** 1 Department of Molecular Pathology, Hiroshima University Graduate School of Biomedical Sciences, Hiroshima, Japan; 2 Department of Surgery, Hiroshima University Graduate School of Biomedical Sciences, Hiroshima, Japan; 3 Division of Genetics, National Cancer Center Research Institute, Tokyo, Japan; Vanderbilt University Medical Center, United States of America

## Abstract

*REG4*, which encodes Reg IV protein, is a member of the calcium-dependent lectin superfamily and potent activator of the epidermal growth factor receptor/Akt/activator protein-1 signaling pathway. Several human cancers overexpress Reg IV, and Reg IV expression is associated with intestinal phenotype differentiation. However, regulation of *REG4* transcription remains unclear. In the present study, we investigated whether CDX2 regulates Reg IV expression in gastric cancer (GC) cells. Expression of Reg IV and CDX2 was analyzed by Western blot and quantitative reverse transcription–polymerase chain reaction in 9 GC cell lines and 2 colon cancer cell lines. The function of the 5′-flanking region of the *REG4* gene was characterized by luciferase assay. In 9 GC cell lines, endogenous Reg IV and CDX2 expression were well correlated. Using an estrogen receptor-regulated form of CDX2, rapid induction of Reg IV expression was observed in HT-29 cells. Reporter gene assays revealed an important role in transcription for consensus CDX2 DNA binding elements in the 5′-flanking region of the *REG4* gene. Chromatin immunoprecipitation assays showed that CDX2 binds directly to the 5′-flanking region of *REG4*. These results indicate that CDX2 protein directly regulates Reg IV expression.

## Introduction

Gastric cancer (GC) is one of the most common human cancers in the world. Cancer develops as a result of multiple genetic and epigenetic alterations [1]. We previously performed serial analysis of gene expression (SAGE) of four primary GCs and identified several GC-specific genes [2]. Of these genes, *regenerating islet-derived family member 4* (*REG4*, which encodes Reg IV protein) is a candidate gene for cancer-specific expression [3]. *REG4* is a member of the *REG* gene family, which belongs to the calcium-dependent lectin superfamily. *REG4* was originally identified by high-throughput sequence analysis of a large inflammatory bowel disease cDNA library [4]. Reg IV is a potent activator of the epidermal growth factor receptor (EGFR)/Akt/activator protein-1 (AP-1) signaling pathway in colon cancer cells and increases expression of Bcl-2, Bcl-xl and survivin, which are proteins associated with the inhibition of apoptosis [5]. Amplification of the *REG4* gene has been reported in pancreatic cancer [6]. Reg IV has been identified as one of the genes up-regulated in cancer-initiating cells [7]. We have previously examined the effect of forced expression of Reg IV in GC cell line. We showed that Reg IV inhibits 5-fluorouracil (5-FU)-induced apoptosis through EGFR activation in GC cells [8]. In contrast, Reg IV-overexpressing cells did not show significant differences in proliferation and invasion activity compared with cells transfected with empty vector [8]. These findings support the notion that Reg IV protein participates in gastric carcinogenesis.

GC can be subdivided into four phenotypes according to mucin expression: gastric or foveolar phenotype; intestinal phenotype; intestinal and gastric mixed phenotype; and neither gastric nor intestinal phenotype [9]. Distinct genetic changes appear to be associated with gastric and intestinal phenotype GC [10]. In our previous observations, Reg IV was expressed in 30% of GC cases and was correlated with intestinal phenotype [11]. A number of immunohistochemical analyses of Reg IV have been reported in human cancers [11]–[20]. In general, these analyses reported that Reg IV is expressed in adenocarcinoma cells displaying an intestinal phenotype. It has been reported that Reg IV expression is induced by GLI1, which is a key transcriptional factor in the Hedgehog signaling pathway [21], or by growth factors such as EGF, transforming growth factor-α (TGF-α), hepatocyte growth factor (HGF), or basic fibroblast growth factor (bFGF) [22]. However, these molecules are unlikely to account for the association between Reg IV expression and intestinal phenotype differentiation.

We have previously found that expression of Reg IV was correlated with CDX2 expression [11]. CDX2 is a mammalian caudal-related intestinal transcription factor and important for the maintenance of intestinal epithelial cells [23], [24]. Several lines of evidence suggest that intestinal metaplasia of the stomach and intestinal phenotype GC are associated with ectopic CDX2 expression [9], [25]. In the present study, we investigated whether CDX2 regulates Reg IV expression in GC and found that CDX2 directly binds to the 5′-flanking region of *REG4* gene and enhances the promoter activity.

## Results

### Reg IV and CDX2 Expression are Correlated in GC Cells

**Figure 1:**
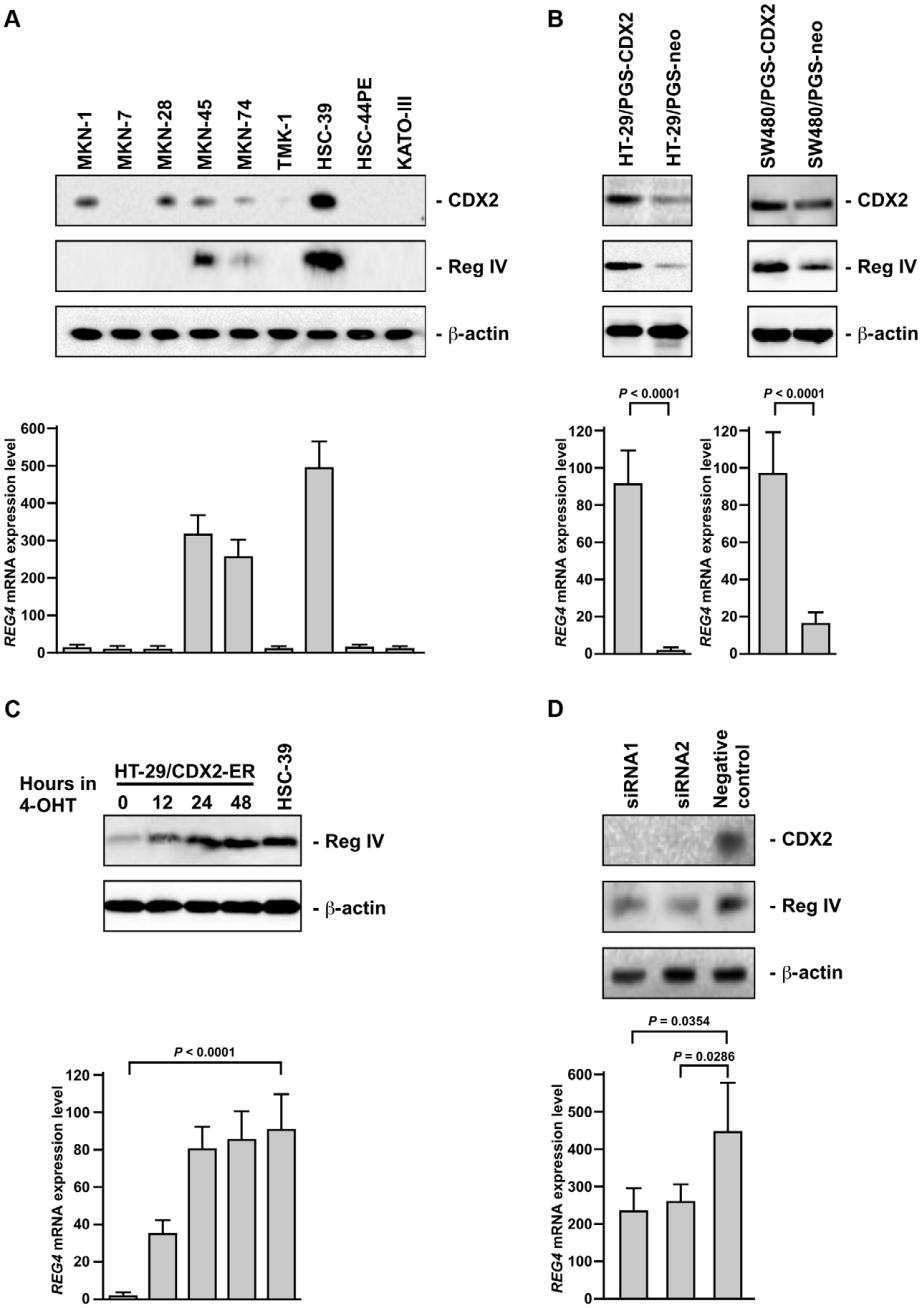
Induction of Reg IV expression by CDX2. A: Western blot analysis of CDX2, Reg IV, and β-actin and qRT-PCR analysis of *REG4* in 9 GC cell lines. B: Western blot analysis of CDX2, Reg IV, and β-actin and qRT-PCR analysis of *REG4* in HT-29/PGS-CDX2, HT-29/PGS-neo, SW480/PGS-CDX2, and SW480/PGS-neo. C: Western blot analysis of Reg IV and β-actin and qRT-PCR analysis of *REG4* in HT-29/CDX2-ER. Time course of *REG4* gene induction in response to activation of a CDX2-ER fusion protein by 4-OHT was analyzed. D: Western blot analysis of CDX2, Reg IV, and β-actin and qRT-PCR analysis of *REG4* in HSC-39 cells transfected with CDX2 siRNA (siRNA1 and siRNA2) and the negative control siRNA. The units of *REG4* mRNA expression level are arbitrary. *P* values were calculated using Student’s t-test. * N.S. = not significant.

We first investigated induction of Reg IV expression by CDX2 in GC cell lines. Western blot analysis of CDX2 in 9 GC cell lines revealed that no or low-level expression of CDX2 was detected in MKN-7, TMK-1, HSC-44PE, and KATO-III (**Fig. 1A**). To determine if CDX2 and Reg IV expression were tightly correlated in GC cells, Western blot and quantitative reverse transcription–polymerase chain reaction (qRT-PCR) analyses of Reg IV were performed on 9 GC cell lines. As shown in **Fig. 1A**, Reg IV protein expression was only detected in the 3 cell lines with high levels of *REG4* transcripts measured by qRT-PCR. Of the 5 GC cell lines with CDX2 protein expression, 2 cell lines (MKN-1 and MKN-28) lacked detectable expression of *REG4* transcripts and protein. The cell lines with undetectable CDX2 protein expression (MKN-7, TMK-1, HSC-44PE, and KATO-III) did not show *REG4* transcripts or protein (**Fig. 1A**).

Next, we generated a polyclonal population of MKN-7, TMK-1, HSC-44PE, and KATO-III cells expressing high levels of CDX2 by infection of the cells with replication-defective retroviruses carrying a full-length human CDX2 cDNA because no or low-level expression of CDX2 was detected in these cell lines. However, overexpression of CDX2 failed to activate Reg IV expression by Western blot (**data not shown**). Because it is possible that CDX2 alone is not sufficient for activating Reg IV expression, expression of *CDH17* (encoding LI-cadherin protein), which is one of the targets of CDX2 [24], was also investigated. However, activation of LI-cadherin expression was not found in MKN-7, TMK-1, HSC-44PE, and KATO-III cells expressing high levels of CDX2 (**data not shown**). Because we showed activation of LI-cadherin expression by CDX2 in the HT-29 colon cancer cell line [24], induction of Reg IV expression was investigated in the same cell line. As shown in **Fig. 1B**, induction of Reg IV expression was detected in HT-29 cells infected with retroviruses carrying a full-length human CDX2 cDNA. We also generated a polyclonal population of SW480 (colon cancer cell line) cells expressing high levels of CDX2 by infection of the cells with replication-defective retroviruses carrying a full-length human CDX2 cDNA. As shown in **Fig. 1B**, induction of Reg IV expression was found in SW480 cells infected with retroviruses carrying a full-length human CDX2 cDNA. These results suggest that Reg IV expression can be induced by CDX2 in cell lines derived from colon cancer. Because in intestinal metaplasia of the stomach, CDX2 and Reg IV expression are well correlated [11], the use of a colon cancer cell line might be suitable for the model of intestinal metaplasia.

To better assess the relationship between CDX2 and Reg IV expression, we studied Reg IV expression in an HT-29-derived line with tightly regulated CDX2 activity. We used a polyclonal HT-29 cell line that had been transduced with the pCDX2-ER vector. The pCDX2-ER vector encodes a chimeric protein in which full-length CDX2 sequences are fused upstream of a mutated estrogen receptor (ER) ligand–binding domain. The mutated ER ligand-binding domain no longer binds estrogen, but retains the ability to bind tamoxifen. Treatment of the HT-29/CDX2-ER cell line with 4-hydroxytamoxifen (4-OHT) resulted in strong induction of Reg IV protein expression within 48 hours (**Fig. 1C**). These results indicate that Reg IV is a direct or primary target gene regulated by CDX2. However, CDX2 alone is not sufficient for activating Reg IV expression.

### Inhibition of CDX2 by RNA Interference (RNAi) Results in the Down-regulation of Reg IV in GC Cells

To determine whether CDX2 is necessary for Reg IV expression in GC cells, we analyzed the effect of inhibiting CDX2 expression by RNAi in the level of Reg IV expression in HSC-39 cell line because high endogenous CDX2 and Reg IV expression was detected in HSC-39 cell line. CDX2-specific small interfering RNAs (siRNAs) significantly suppressed CDX2 protein expression 3 days after transfection, and expression of Reg IV transcript was down-regulated approximately 50% by CDX2 siRNAs in HSC-39 compared with its levels in control siRNA-treated cells (**Fig. 1D**). These results indicate that CDX2 is involved in maintaining Reg IV gene expression.

### Functional Characterization the 5′-flanking Region of *REG4* Gene by Luciferase Assay

**Figure 2:**
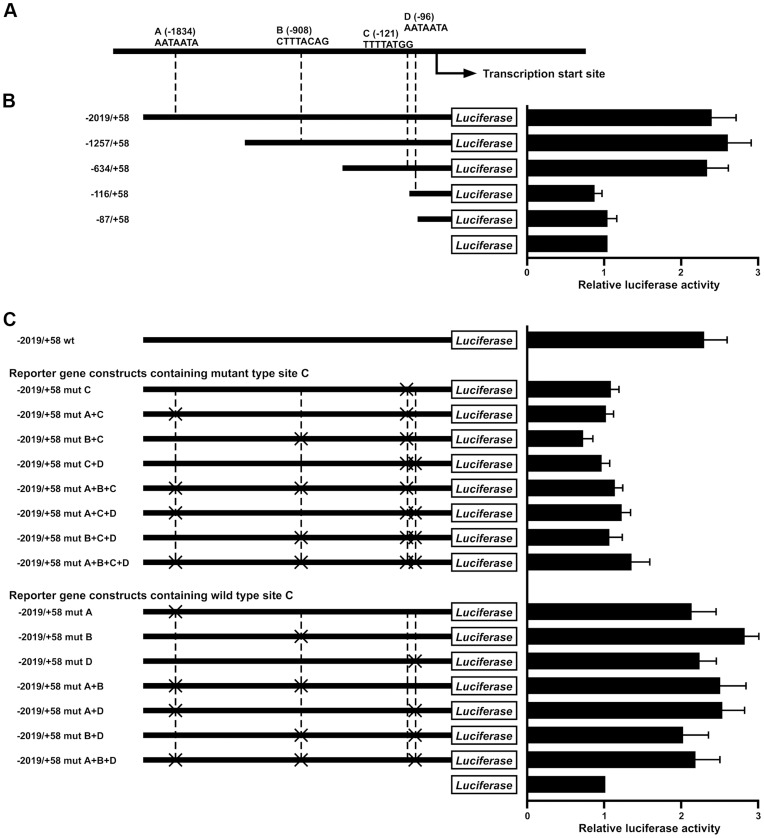
*REG4* promoter analysis. Localization of regulatory elements and CDX2 binding sites in the 5′-flanking region of the *REG4* gene. A: Schematic representation of the 5′-flanking region of the *REG4* gene. The location and sequence of 4 consensus CDX2-binding sites in the 5′-flanking region of *REG4* (i.e., sites A, B, C, and D) is indicated. B: Schematic representation of *REG4* reporter gene constructs. The *REG4* genomic DNA sequences present in the reporter gene vectors are indicated. Key sequences for *REG4* transcription reside between base pairs −634 and −116. Reporter assays with the series of *REG4* deletion constructs were performed in the CDX2-expressing GC cell line, HSC-39. The luciferase activity of the empty pGL4.10 basic vector was assigned a value of 1. The reporter assays were performed in triplicate, and mean and SD values of luciferase activity are shown. C: Localized mutations in the candidate CDX2-binding sites (i.e., sites A, B, C, and D) were introduced into the −2019/+58 construct, and the series of constructs generated is shown. The CDX2 candidate binding site designated as “C” plays critical roles in *REG4* transcription. Reporter assays were performed in CDX2-expressing GC cell line, HSC-39. The activity of the pGL4.10 basic vector was assigned a value of 1. Assays were performed in triplicate. Mean and SD luciferase activity values are shown.

To identify potential CDX2-binding sites in the *REG4* promoter region, a search of the genomic sequences immediately 5′ to the presumptive transcription start site was performed, using a consensus binding element for the CdxA chicken *caudal* homologue (5′-A, A/T, T, A/T, A, T, A/G-3′) [26] and a previously described search algorithm [27]. We found four putative CDX2-binding sites in the 2 kilobase (kb) 5′-flanking region of the *REG4* gene (**Fig. 2A**). These were: site A (5′-AATAATA-3′, from −1828 to −1834), site B (5′-CTTTACAG-3′, from −901 to −908), site C (5′-TTTTATGG-3′, from −114 to −121), site D (5′- AATAATA -3′, from −90 to −96). To assess the role of these presumptive CDX2-binding sites in regulating *REG4* transcription, various reporter gene constructs were generated. As shown in **Fig. 2B**, reporter gene constructs containing 2.1, 1.2, or 0.6 kb of 5′-flanking sequence from the *REG4* gene showed strong activity in the HSC-39 cells, which display strong endogenous expression of *REG4* transcripts and protein. By comparison, MKN-1 cells have little endogenous *REG4* transcript and displayed little or no transcriptional activity induced by the 2.1, 1.2, or 0.6 kb *REG4* reporter gene constructs (**data not shown**). The *REG4* reporter gene constructs containing base pairs −116 to +58 and −87 to +58 had reduced activity in the HSC-39 cells (**Fig. 2B**), indicating that sequences between base pairs −634 and −116 play a key role in activating *REG4* transcription. Furthermore, we analyzed single and multiple mutations in the presumptive CDX2-binding sites in the 5′-flanking region of the *REG4* gene using HSC-39 cells (**Fig. 2C**). As expected, presumptive CDX2-binding site C, which is located between base pairs −634 and −116, plays a crucial role in activating *REG4* transcription.

### CDX2 Directly Binds to the 5′-flanking Region of *REG4* Gene

**Figure 3:**
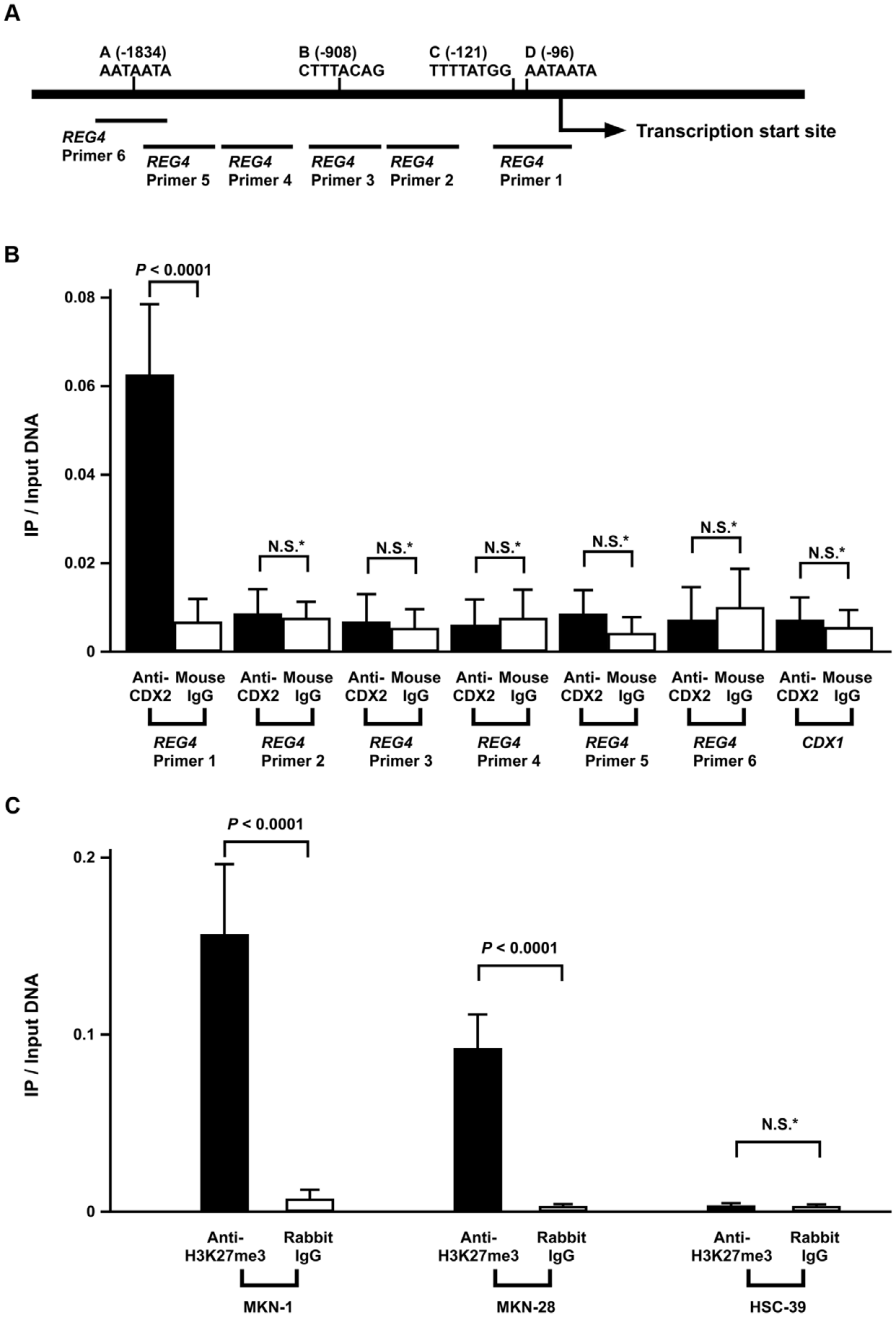
ChIP analysis of the 5′-flanking region of the *REG4* gene. A: Schematic representation of the 5′-flanking region of the *REG4* gene. The location of 4 consensus CDX2-binding sites in the 5′-flanking region of *REG4* (sites A, B, C, and D) and PCR primers (*REG4* Primer 1, 2, 3, 4, 5, and 6) are indicated. B: CDX2 binding to *REG4* promoter region shown by ChIP. Bulk (input) DNA was prepared as well as DNA isolated from ChIP with anti-CDX2 monoclonal antibody or mouse IgG. qPCRs were performed in triplicate for each sample primer set, and the mean and SD of the three experiments was calculated. C: ChIP analysis of H3K27me3 enrichment in the *REG4* gene promoter. ChIP enrichment was measured using qPCR. qPCRs were performed in triplicate for each sample primer set, and the mean and SD of the three experiments was calculated. *P* values were calculated using Student′s t-test. * N.S. = not significant.

To analyze whether CDX2 directly binds to the putative CDX2-binding sites in the *REG4* 5′-flanking region, we performed chromatin immunoprecipitation (ChIP) assays using HSC-39 cells. Using 6 primers for the *REG4* 5′-flanking region (**Fig. 3A**), we recovered DNA fragments containing the *REG4* 5′-flanking region by primer 1, which encompasses presumptive CDX2-binding site C (**Fig. 3B**). DNA fragments from the 5′-flanking region of *REG4*, which were generated using primers 2, 3, 4, 5 and 6 such that they did not contain presumptive CDX2 binding sites, were not recovered by the anti-CDX2 antibody. The specificity of recovery of the *REG4* promoter region following ChIP with anti-CDX2 antibody was shown by the fact that other irrelevant DNA fragments lacking CDX2-binding sites (e.g., exon 3 of the *CDX1* gene) were not recovered (**Fig. 3B**). In addition, mock immunoprecipitation (mouse IgG) yielded few *REG4* or *CDX1*-specific DNA fragments (**Fig. 3B**). All these findings suggest that CDX2 activates *REG4* transcription by directly binding to sequences in the 5′-flanking region of the gene.

### Trimethylation of Histone H3 Lysine 27 (H3K27me3) on the *REG4* Promoter in the GC Cell Lines

Although CDX2 protein expression was found in MKN-1 and MKN-28 cell lines, these 2 cell lines lacked detectable expression of *REG4* transcript and protein. Because it has been reported that DNA hypermethylation of CpG islands is associated with silencing of several genes [28], we investigate whether DNA methylation induced transcriptional inactivation of Reg IV in MKN-1 and MKN-28 cells. We treated these cells with a demethylating agent, 5-aza-2′-deoxycytidine (Aza-dC) and then performed qRT-PCR. However, Reg IV expression was not restored in these cell lines (**data not shown**), suggesting that DNA methylation is not likely to affect Reg IV expression. It has been also reported that H3K27me3 has been associated with repressed gene expression [29]. We further investigated H3K27me3 in GC cell lines. To determine the enrichment of H3K27me3 on the *REG4* promoter in the GC cell lines, ChIP assays were performed. In MKN-1 and MKN-28 cell lines, H3K27me3 levels on the *REG4* promoter region were high, whereas in HSC-39 cell line, H3K27me3 level on the *REG4* promoter region was low (**Fig. 3C**). These results suggest that closed chromatin structure of *REG4* promoter can inhibit Reg IV expression by CDX2.

## Discussion

Although it has been reported that Reg IV expression is induced by GLI1 [21] or EGF [22], these molecules are unlikely to account for the association between Reg IV and intestinal differentiation. In the present study, we showed that endogenous CDX2 and Reg IV expression were well correlated in GC cell lines. In addition, using an ER-regulated form of CDX2, we found that there was rapid induction of Reg IV expression after 4-OHT treatment. Reporter gene assays revealed an important role for consensus CDX2 DNA binding elements in the *REG4* promoter region in its transcription. Subsequent ChIP assays showed that CDX2 binds directly to the *REG4* promoter. We previously showed that in primary GC tissue and intestinal metaplasia of the stomach, CDX2 and Reg IV expression were well correlated [11]. These results indicate that CDX2 protein directly regulates Reg IV expression in GC and intestinal metaplasia of the stomach.

CDX2 is overexpressed in intestinal phenotype GC and in intestinal metaplasia of the stomach [9], [25]. In contrast, loss of CDX2 expression was observed in a subset of primary colorectal cancers, usually in poorly differentiated colorectal cancers [30]. The significance of alteration of CDX2 expression in human cancers remains unclear, and therefore it is important to define the target genes which are downstream of CDX2. We have identified several CDX2-regulated genes such as *CDH17* (which encodes LI-cadherin) [24], *HEPH* (which encodes hephaestin) [31], *ABCB1* (which encodes multidrug resistance 1) [32], and *DSC2* (which encodes desmocollin 2) [33]. Among these genes, *ABCB1* was originally identified as an overexpressed and amplified gene in multiple drug-resistant cells, and its product, P-glycoprotein, seems to play a critical role in drug resistance [34]. Previously, we reported that forced expression of Reg IV in GC cells inhibited 5-FU-induced apoptosis through induction of Bcl-2 and dihydropyrimidine dehydrogenase [8]. Taken together, it is possible that in intestinal phenotype GC, expression (or ectopic expression) of CDX2 induces Reg IV and multidrug resistance 1 expression, resulting in an increase in drug-resistance. In fact, it has been reported that postoperative chemotherapy is not beneficial for patients with intestinal phenotype GC [35].

Although our data support the view that CDX2 plays a role in regulating *REG4* transcription via binding to the promoter region, several findings indicate that CDX2 alone is not sufficient for activating *REG4* expression. In the present study, we generated a polyclonal population of MKN-7, TMK-1, HSC-44PE, and KATO-III cells which express high levels of CDX2 by infection with retroviruses carrying a full-length human CDX2 cDNA. However, overexpression of CDX2 failed to activate Reg IV expression. In GC cell lines, none of the cell lines with undetectable CDX2 protein expression had detectable *REG4* transcripts and protein. Therefore, CDX2 is required for Reg IV expression, but CDX2 alone is not sufficient for activating Reg IV expression. In the present study, nine GC cell lines were studied. The origins of the cell lines were as follows. The MKN-7, MKN-28, and MKN-74 cell lines were established from intestinal type GC. The TMK-1 and MKN-45 cell lines were established from diffuse type GC. The KATO-III, HSC-39, and HSC-44PE cell lines were established from signet ring cell carcinoma. The MKN-1 cell line was established from adenosquamous cell carcinoma. Because in intestinal metaplasia of the stomach, CDX2 and Reg IV expression are well correlated, GC cell lines established from diffuse type GC or signet ring cell carcinoma may not be suitable for the analysis of Reg IV induction by CDX2. In fact, Reg IV expression can be induced by CDX2 in cell lines derived from colon cancer in the present study. Furthermore, we showed that H3K27me3 levels on the *REG4* promoter region were high in MKN-1 and MKN-28 GC cell lines. These 2 cell lines lacked detectable expression of *REG4* although CDX2 protein expression was found. Therefore, H3K27me3 levels on the *REG4* promoter region may be high in MKN-7, TMK-1, HSC-44PE, and KATO-III cells, in which overexpression of CDX2 failed to activate Reg IV expression.

It has been reported that *REG4* mRNA expression was enhanced by stimulation with TGF-α, EGF, HGF, or bFGF through activation of the mitogen-activated protein kinase (MAPK) pathway [22]. Thus, it could be hypothesized that Reg IV is also regulated by downstream transcriptional factors of MAPK pathways. We performed *in silico* analyses of the *REG4* gene 5′-flanking region, and found at least one presumptive AP-1 consensus sequences (at −883 base pairs of *REG4* gene 5′-flanking region), which is a downstream transcriptional factor of MAPK signalling. In the present study, HSC-39 cells showed similar transcriptional activity of reporter gene constructs containing 1.2 kb and 0.6 kb of *REG4* 5′-flanking sequence. As the effect of EGF or TGF-α on *REG4* transcription was not investigated in the present study, further investigation is needed to clarify the signalling mechanisms which induce regulation of *REG4* transcription.

In conclusion, our present data show that CDX2 protein directly regulates Reg IV expression. Reg IV activates the EGFR/Akt/AP-1 signaling pathway. As intestinal phenotype GC frequently expresses EGFR [36], it is suggested that this Reg IV-activated pathway plays an important role in this subtype of GC. Because CDX2 also induces expression of the multidrug resistance gene, *ABCB1*, anti-EGFR therapy but not chemotherapy may be beneficial for patients with intestinal phenotype GC.

## Materials and Methods

### Plasmids

The CDX2 cDNA was inserted into the multiple cloning site of the retroviral expression vector pPGS-CMV-CITE-neo as described previously [24]. The full-length, wild-type CDX2 cDNA was also subcloned into the retroviral vector pBabe-Puro ER as described previously to generate pCDX2-ER [24]. The pCDX2-ER vector encodes a chimeric protein in which full-length CDX2 sequences are fused upstream of a mutated ER ligand–binding domain. The mutated ER ligand-binding domain no longer binds estrogen, but retains the ability to bind tamoxifen. Genomic DNA sequences from the 5′-flanking region of the human *REG4* gene were amplified by PCR using genomic DNA purified from HSC-39 cells as a template and subcloned into the pGL4.10 [luc2] vector (Promega, Madison, WI). PCR-based approaches were used to introduce mutations into the presumptive CDX2-binding sites in the pGL4.10-REG4 reporter gene construct using QuikChange Site-Directed Mutagenesis Kit (Stratagene, La Jolla, CA). Four putative CDX2-binding sites were changed. All fragments generated by PCR were verified by automated sequencing. The plasmid pGL4.74 [hRluc/TK] vector (Promega) was used as a control for transfection efficiency in reporter assays.

### Cell Lines, Retrovirus Infections, and Drug Treatment

The amphotropic Phoenix packaging cell line was provided by G. Nolan (Stanford University, Stanford, CA) [37]. Nine cell lines derived from human GC and 2 cell lines derived from human colon cancer were used. The TMK-1 cell line was established in our laboratory [38]. The HSC-39 and HSC-44PE cell lines were established by one of the authors (Kazuyoshi Yanagihara) [39], [40]. Five GC cell lines of the MKN series were kindly provided by Dr. Toshimitsu Suzuki [41], [42]. The KATO-III cell line was kindly provided by Dr. Morimasa Sekiguchi [43]. The HT-29 and SW480 colon cancer cell lines were obtained from the American Type Culture Collection. Cells were stored in liquid nitrogen until the initiation of this study. After thawing from frozen stock, the cells were kept at low passage throughout the study. Consistent cell morphology was monitored by comparison of microscopic images. The Phoenix packaging cells were transfected with retroviral expression constructs (pPGS-CDX2, pPGS-neo, and pCDX2-ER) and the supernatant containing nonreplicating amphotropic virus was harvested as previously described [24]. In HT-29 cells expressing the CDX2-ER fusion protein (HT-29/CDX2-ER), CDX2 function was activated by addition of 4-hydroxytamoxifen (4-OHT) (Sigma Chemical, St. Louis, MO) to the growth medium at a final concentration of 500 nmol. To investigate whether DNA methylation induced transcriptional inactivation of Reg IV, cells were treated with a final concentration of 1 µM Aza-dC (Sigma Chemical) for 5 days before they were harvested for RNA extraction.

### Western Blot Analysis

For Western blot analysis, cells were lysed as described previously [44]. Protein concentrations were determined by Bradford protein assay (BioRad, Richmond, CA) with BSA used as the standard. The lysates (20 µg) were solubilized in Laemmli’s sample buffer by boiling and then subjected to 12% SDS-polyacrylamide gel electrophoresis followed by electro-transfer onto a nitrocellulose filter. The filter was incubated for 1 hour at room temperature with an anti-Reg IV antibody (rabbit polyclonal antibody developed in our laboratory, Ref. 10) or anti-CDX2 antibody (BioGenex, San Ramon, CA). Peroxidase-conjugated anti-rabbit or anti-mouse IgG was used in the secondary reaction. Immunocomplexes were visualized with an ECL Plus Western Blot Detection System (Amersham Biosciences, Piscataway, NJ). β-actin (Sigma Chemical) was also detected as a loading control.

### qRT-PCR Analysis

Total RNA was extracted with an RNeasy Mini Kit (Qiagen, Valencia, CA), and 1 µg of total RNA was converted to cDNA with a First Strand cDNA Synthesis Kit (Amersham Biosciences). Quantitation of *REG4* mRNA levels was performed by real-time fluorescence detection as described previously [45]. PCR was performed with a SYBR Green PCR Core Reagents Kit (Applied Biosystems, Foster City, CA). Real-time detection of the emission intensity of SYBR green bound to double-stranded DNA was performed with an ABI PRISM 7900 Sequence Detection System (Applied Biosystems) as described previously [46]. *ACTB*-specific PCR products were amplified from the same RNA samples and served as an internal control. Sequences of primers for *REG4* qRT-PCR are shown in **Table 1**. qRT-PCRs were performed in triplicate for each sample primer set, and the mean and standard deviation (SD) of the three experiments was calculated as the relative quantification value. At the end of 40 PCR cycles, reaction products were separated electrophoretically on 8% non-denaturing polyacrylamide gels for visual confirmation of PCR products.

**Table 1:** Primer sequences for qRT-PCR and ChIP assay.

Sense	Anti-sense
qRT-PCR for *REG4*	
5′-GCCCCGCCATCCCTT-3′	5′-CTGCTCGAGACAGCCAGAGA-3′
qRT-PCR for *ACTB*	
5′-TCACCGAGCGCGGCT-3′	5′-TAATGTCACGCACGATTTCCC-3′
ChIP for *REG4* (Primer 1)	
5′-GGAGAGGTTCTTTTCCTGGCTAG-3′	5′-GCAACCAAGACTCTAAGGGCC-3′
ChIP for *REG4* (Primer 2)	
5′-CCCTTTGCCATCTATACTGGAAA-3′	5′-CATTACACACTCAAGAAACCCAACC-3′
ChIP for *REG4* (Primer 3)	
5′-TCAGCTTCACCCACAACTGTCT-3′	5′-TTAGGTTGTAGGTGCCAGAGATGA-3′
ChIP for *REG4* (Primer 4)	
5′-CAAAGGTTTATGTGAGTCCTATCAATG-3′	5′-CCTGTGTTTCCAGCAGCCAT-3′
ChIP for *REG4* (Primer 5)	
5′-CTATTCGAAAGCTGCCTGGC-3′	5′-AAATTGTCTGAATCAAAAAGGTCCA-3′
ChIP for *REG4* (Primer 6)	
5′-GCAGGAGATAAAAGGCTACACGTT-3′	5′-GGAGAGATAAAGTGGAAGCCAGG-3′
ChIP for *CDX1*	
5′-TCCTCGTCTCTCCTTCTTGC-3′	5′-AGAAGGTCAGGGCTGAGACTC-3′

### RNAi

To knockdown the endogenous CDX2, RNAi was performed. Two siRNA duplexes targeting CDX2 (5′-AACCAGGACGAAAGACAAAUA-3′, CDX2 siRNA1; and 5′-AAGCCUCAGUGUCUGGCUCUG-3′, CDX2 siRNA2) and a nonsilencing siRNA duplex (5′-AAUUCUCCGAACGUGUCACGU-3′) were synthesized (Qiagen). Transfection was performed using Lipofectamine RNAiMAX (Invitrogen, Carlsbad, CA) according to the manufacturer’s protocol. Briefly, 60 pmol of siRNA and 10 µL of Lipofectamine RNAiMAX were mixed in 1 mL of RPMI medium (10 nmol/L final siRNA concentration). After 20 min of incubation, the mixture was added to the cells and these were plated on dishes for each assay. Three days after transfection, cells were analyzed for all experiments.

### Reporter Gene Assays

HSC-39 and MKN-1 cells were seeded in 6-well plates (BD Falcon, Franklin Lakes, NJ). Transfection of cells at 50%–80% confluency was performed with 3 µL of FuGENE6 Transfection Reagent (Roche Diagnostics, Indianapolis, IN), 0.8 µg of pGL4.10 reporter gene constructs, and 0.2 µg pGL4.74 [hRluc/TK] vector (Promega). At 48 hours after transfection, cells were collected and resuspended in passive lysis buffer (Promega). Luciferase activity was determined with a dual luciferase assay system (GloMax 96 Microplate Luminometer, Promega).

### ChIP Assays

The ChIP assays were performed using the EZ-ChIP Chromatin Immunoprecipitation Kit (Millipore, Billerica, MA) per manufacture instructions. To analyze whether CDX2 directly binds to the putative CDX2-binding sites in the *REG4* 5′-flanking region, we performed ChIP assays using HSC-39 cells. In brief, HSC-39 cells (1–2×10^7^) were cross linked with 1% formaldehyde in phosphate buffered saline (PBS) for 15 min at 37°C, and glycine was added to quench reactive aldehydes. After washing cells with cold PBS, cells were resuspended in SDS lysis buffer (1% SDS, 10 mM EDTA, and 50 mM Tris pH 8.1) with Proteinase Inhibitor (Roche Diagnostics). After samples were sonicated, chromatin extracts containing DNA fragments (average size, 500 base pairs) were immunoprecipitated using 2 µg monoclonal anti-CDX2 antibody (BioGenex) or 2 µg mouse IgG (Millipore). Each immunoprecipitated DNA sample was quantified by qPCR using primers listed in **Table 1**. As a negative control, an approximately 200 base pairs DNA fragment from exon 3 of the *CDX1* gene was amplified by PCR using specific primers (**Table 1**).

To determine the enrichment of H3K27me3 on the *REG4* promoter in the GC cell lines, ChIP assays were performed using MKN-1, MKN-28, and HSC-39 cell lines. In brief, GC cells (1–2×10^7^) were cross linked with 1% formaldehyde in PBS for 15 min at 37°C, and glycine was added to quench reactive aldehydes. After washing cells with cold PBS, cells were resuspended in SDS lysis buffer with Proteinase Inhibitor (Roche Diagnostics). After samples were sonicated, chromatin extracts containing DNA fragments (average size, 500 base pairs) were immunoprecipitated using 2 µg polyclonal anti-H3K27me3 antibody (Abcam, Cambridge, MA) or 2 µg rabbit IgG (Millipore). Each immunoprecipitated DNA sample was quantified by qPCR using *REG4* Primer 1 (**Table 1**).

qPCRs were performed in triplicate for each sample primer set, and the mean and standard deviation (SD) of the three experiments was calculated as the relative quantification value. At the end of 40 PCR cycles, reaction products were separated electrophoretically on 8% non-denaturing polyacrylamide gels for visual confirmation of PCR products.

## References

[1] Yasui W, Sentani K, Sakamoto N, Anami K, Naito Y, et al (2011) Molecular pathology of gastric cancer: research and practice. Pathol Res Pract 207: 608–12. 22005013 10.1016/j.prp.2011.09.006

[2] Oue N, Hamai Y, Mitani Y, Matsumura S, Oshimo Y, et al (2004) Gene expression profile of gastric carcinoma: identification of genes and tags potentially involved in invasion, metastasis, and carcinogenesis by serial analysis of gene expression. Cancer Res 64: 2397–405. 15059891 10.1158/0008-5472.can-03-3514

[3] Aung PP, Oue N, Mitani Y, Nakayama H, Yoshida K, et al (2006) Systematic search for gastric cancer-specific genes based on SAGE data: melanoma inhibitory activity and matrix metalloproteinase-10 are novel prognostic factors in patients with gastric cancer. Oncogene 25: 2546–57. 16331256 10.1038/sj.onc.1209279

[4] Hartupee JC, Zhang H, Bonaldo MF, Soares MB, Dieckgraefe BK (2001) Isolation and characterization of a cDNA encoding a novel member of the human regenerating protein family: Reg IV. Biochim Biophys Acta 1518: 287–93. 11311942 10.1016/s0167-4781(00)00284-0

[5] Bishnupuri KS, Luo Q, Murmu N, Houchen CW, Anant S, et al (2006) Reg IV activates the epidermal growth factor receptor/Akt/AP-1 signaling pathway in colon adenocarcinomas. Gastroenterology 130: 137–49. 16401477 10.1053/j.gastro.2005.10.001

[6] Legoffic A, Calvo E, Cano C, Folch-Puy E, Barthet M, et al (2009) The reg4 gene, amplified in the early stages of pancreatic cancer development, is a promising therapeutic target. PLoS One 4: e7495. 19834624 10.1371/journal.pone.0007495 PMC2760775

[7] Katsuno Y, Ehata S, Yashiro M, Yanagihara K, Hirakawa K, et al.. (2012) Coordinated expression of REG4 and aldehyde dehydrogenase 1 regulating tumourigenic capacity of diffuse-type gastric carcinoma-initiating cells is inhibited by TGF-b. J Pathol, in press. 10.1002/path.4020 22430847

[8] Mitani Y, Oue N, Matsumura S, Yoshida K, Noguchi T, et al (2007) Reg IV is a serum biomarker for gastric cancer patients and predicts response to 5-fluorouracil-based chemotherapy. Oncogene 26: 4383–93. 17237819 10.1038/sj.onc.1210215

[9] Tatematsu M, Tsukamoto T, Inada K (2003) Stem cells and gastric cancer: role of gastric and intestinal mixed intestinal metaplasia. Cancer Sci 94: 135–41. 12708487 10.1111/j.1349-7006.2003.tb01409.x PMC11160206

[10] Motoshita J, Oue N, Nakayama H, Kuraoka K, Aung PP, et al (2005) DNA methylation profiles of differentiated-type gastric carcinomas with distinct mucin phenotypes. Cancer Sci 96: 474–9. 16108828 10.1111/j.1349-7006.2005.00074.x PMC11158929

[11] Oue N, Mitani Y, Aung PP, Sakakura C, Takeshima Y, et al (2005) Expression and localization of Reg IV in human neoplastic and non-neoplastic tissues: Reg IV expression is associated with intestinal and neuroendocrine differentiation in gastric adenocarcinoma. J Pathol 207: 185–98. 16086444 10.1002/path.1827

[12] Takehara A, Eguchi H, Ohigashi H, Ishikawa O, Kasugai T, et al (2006) Novel tumor marker REG4 detected in serum of patients with resectable pancreatic cancer and feasibility for antibody therapy targeting REG4. Cancer Sci 97: 1191–7. 16918991 10.1111/j.1349-7006.2006.00297.x PMC11159249

[13] Nakata K, Nagai E, Ohuchida K, Aishima S, Hayashi A, et al (2009) REG4 is associated with carcinogenesis in the ‘intestinal’ pathway of intraductal papillary mucinous neoplasms. Mod Pathol 22: 460–8. 19136934 10.1038/modpathol.2008.205

[14] Oue N, Kuniyasu H, Noguchi T, Sentani K, Ito M, et al (2007) Serum concentration of Reg IV in patients with colorectal cancer: overexpression and high serum levels of Reg IV are associated with liver metastasis. Oncology 72: 371–80. 18187959 10.1159/000113147

[15] Ohara S, Oue N, Matsubara A, Mita K, Hasegawa Y, et al (2008) Reg IV is an independent prognostic factor for relapse in patients with clinically localized prostate cancer. Cancer Sci 99: 1570–7. 18754868 10.1111/j.1349-7006.2008.00846.x PMC11158611

[16] Sasahira T, Oue N, Kirita T, Luo Y, Bhawal UK, et al (2008) Reg IV expression is associated with cell growth and prognosis of adenoid cystic carcinoma in the salivary gland. Histopathology 53: 667–75. 19076683 10.1111/j.1365-2559.2008.03188.x

[17] Hayashi T, Matsubara A, Ohara S, Mita K, Hasegawa Y, et al (2009) Immunohistochemical analysis of Reg IV in urogenital organs: Frequent expression of Reg IV in prostate cancer and potential utility as serum tumor marker. Oncol Rep 21: 95–100. 19082448

[18] Tamura H, Ohtsuka M, Washiro M, Kimura F, Shimizu H, et al (2009) Reg IV expression and clinicopathologic features of gallbladder carcinoma. Hum Pathol 40: 1686–92. 19716164 10.1016/j.humpath.2009.06.001

[19] Sentani K, Oue N, Noguchi T, Sakamoto N, Matsusaki K, et al (2010) Immunostaining of gastric cancer with neuroendocrine differentiation: Reg IV-positive neuroendocrine cells are associated with gastrin, serotonin, pancreatic polypeptide and somatostatin. Pathol Int 60: 291–7. 20403031 10.1111/j.1440-1827.2010.02519.x

[20] Heiskala K, Arola J, Heiskala M, Andersson LC (2010) Expression of Reg IV and Hath1 in neuroendocrine neoplasms. Histol Histopathol 25: 63–72. 19924642 10.14670/HH-25.63

[21] Wang F, Xu L, Guo C, Ke A, Hu G, et al (2011) Identification of RegIV as a novel GLI1 target gene in human pancreatic cancer. PLoS One 6: e18434. 21494603 10.1371/journal.pone.0018434 PMC3073946

[22] Nanakin A, Fukui H, Fujii S, Sekikawa A, Kanda N, et al (2007) Expression of the REG IV gene in ulcerative colitis. Lab Invest 87: 304–14. 17260007 10.1038/labinvest.3700507

[23] Silberg DG, Swain GP, Suh ER, Traber PG (2000) Cdx1 and cdx2 expression during intestinal development. Gastroenterology 119: 961–71. 11040183 10.1053/gast.2000.18142

[24] Hinoi T, Lucas PC, Kuick R, Kuick R, Hanash S, et al (2002) CDX2 regulates liver intestine-cadherin expression in normal and malignant colon epithelium and intestinal metaplasia. Gastroenterology 123: 1565–77. 12404231 10.1053/gast.2002.36598

[25] Almeida R, Silva E, Santos-Silva F, Silberg DG, Wang J, et al (2003) Expression of intestine-specific transcription factors, CDX1 and CDX2, in intestinal metaplasia and gastric carcinomas. J Pathol 199: 36–40. 12474224 10.1002/path.1246

[26] Margalit Y, Yarus S, Shapira E, Gruenbaum Y, Fainsod A (1993) Isolation and characterization of target sequences of the chicken CdxA homeobox gene. Nucleic Acids Res 21: 4915–22. 7909943 10.1093/nar/21.21.4915 PMC311406

[27] Heinemeyer T, Wingender E, Reuter I, Hermjakob H, Kel AE, et al (1998) Databases on transcriptional regulation: TRANSFAC, TRRD and COMPEL. Nucleic Acids Res 26: 362–7. 9399875 10.1093/nar/26.1.362 PMC147251

[28] Jones PA, Baylin SB (2002) The fundamental role of epigenetic events in cancer. Nat Rev Genet 3: 415–428. 12042769 10.1038/nrg816

[29] Mossman D, Scott RJ (2011) Long term transcriptional reactivation of epigenetically silenced genes in colorectal cancer cells requires DNA hypomethylation and histone acetylation. PLoS One 6: e23127. 21829702 10.1371/journal.pone.0023127 PMC3150411

[30] Hinoi T, Tani M, Lucas PC, Caca K, Dunn RL, et al (2001) Loss of CDX2 expression and microsatellite instability are prominent features of large cell minimally differentiated carcinomas of the colon. Am J Pathol 159: 2239–48. 11733373 10.1016/S0002-9440(10)63074-X PMC1850596

[31] Hinoi T, Gesina G, Akyol A, Kuick R, Hanash S, et al (2005) CDX2-regulated expression of iron transport protein hephaestin in intestinal and colonic epithelium. Gastroenterology 128: 946–61. 15825077 10.1053/j.gastro.2005.01.003

[32] Takakura Y, Hinoi T, Oue N, Sasada T, Kawaguchi Y, et al (2010) CDX2 regulates multidrug resistance 1 gene expression in malignant intestinal epithelium. Cancer Res 70: 6767–78. 20699370 10.1158/0008-5472.CAN-09-4701 PMC3153948

[33] Anami K, Oue N, Noguchi T, Sakamoto N, Sentani K, et al (2010) Search for transmembrane protein in gastric cancer by the Escherichia coli ampicillin secretion trap: expression of DSC2 in gastric cancer with intestinal phenotype. J Pathol 221: 275–84. 20527021 10.1002/path.2717

[34] Pastan I, Gottesman MM (1991) Multidrug resistance. Annu Rev Med 42: 277–86. 2035973 10.1146/annurev.me.42.020191.001425

[35] Tajima Y, Shimoda T, Nakanishi Y, Yokoyama N, Tanaka T, et al (2003) Association of gastric and intestinal phenotypic marker expression of gastric carcinomas with tumor thymidylate synthase expression and response to postoperative chemotherapy with 5-fluorouracil. J Cancer Res Clin Oncol 129: 683–90. 14576935 10.1007/s00432-003-0476-0 PMC12161923

[36] Motoshita J, Nakayama H, Taniyama K, Matsusaki K, Yasui W (2006) Molecular characteristics of differentiated-type gastric carcinoma with distinct mucin phenotype: LI-cadherin is associated with intestinal phenotype. Pathol Int 56: 200–5. 16634965 10.1111/j.1440-1827.2006.01946.x

[37] Grignani F, Kinsella T, Mencarelli A, Valtieri M, Riganelli D, et al (1998) High-efficiency gene transfer and selection of human hematopoietic progenitor cells with a hybrid EBV/retroviral vector expressing the green fluorescence protein. Cancer Res 58: 14–19. 9426049

[38] Ochiai A, Yasui W, Tahara E (1985) Growth-promoting effect of gastrin on human gastric carcinoma cell line TMK-1. Jpn J Cancer Res 76: 1064–71. 3003017

[39] Yanagihara K, Seyama T, Tsumuraya M, Kamada N, Yokoro K (1991) Establishment and characterization of human signet ring cell gastric carcinoma cell lines with amplification of the c-myc oncogene. Cancer Res 51: 381–6. 1846312

[40] Yanagihara K, Tanaka H, Takigahira M, Ino Y, Yamaguchi Y, et al (2004) Establishment of two cell lines from human gastric scirrhous carcinoma that possess the potential to metastasize spontaneously in nude mice. Cancer Sci 95: 575–82. 15245593 10.1111/j.1349-7006.2004.tb02489.x PMC11159459

[41] Hoji H (1977) Establishiment of cultured cell lines of human stomach cancer – origin and their morphological characteristics. Niigata Igakukai Zasshi 91: 737–752.

[42] Motoyama T, Hoji H, Watanabe H (1986) Comparison of seven cell lines derived from human gastric carcinomas. Acta Pathol. Jpn. 36: 65–83. 10.1111/j.1440-1827.1986.tb01461.x 3962675

[43] Sekiguchi M, Sakakibara K, Fuji G (1978) Establishment of cultured cell lines derived from a human gastric carcinoma. Jpn. J. Exp. Med. 48: 61–68. 209229

[44] Yasui W, Ayhan A, Kitadai Y, Nishimura K, Yokozaki H, et al (1993) Increased expression of p34cdc2 and its kinase activity in human gastric and colonic carcinomas. Int J Cancer 53: 36–41. 8416202 10.1002/ijc.2910530108

[45] Gibson UE, Heid CA, Williams PM (1996) A novel method for real time quantitative RT-PCR. Genome Res 6: 995–1001. 8908519 10.1101/gr.6.10.995

[46] Kondo T, Oue N, Yoshida K, Mitani Y, Naka K, et al (2004) Expression of POT1 is associated with tumor stage and telomere length in gastric carcinoma. Cancer Res 64: 523–9. 14744765 10.1158/0008-5472.can-03-1196

